# Causes of death after oral cancer diagnosis: a population based study

**DOI:** 10.3389/fonc.2024.1481688

**Published:** 2024-11-12

**Authors:** Zhenyu Jiang, Li Shao, Jie Zhou, Xuwen Shao, Chao Shen

**Affiliations:** ^1^ Department of Nephrology, The First People’s Hospital of Huzhou, The First Affiliated Hospital of Huzhou Teachers College, Huzhou, China; ^2^ Department of Stomatology, The First People’s Hospital of Huzhou, The First Affiliated Hospital of Huzhou Teachers College, Huzhou, China; ^3^ Department of Oncology, Huzhou Central Hospital, Affiliated Central Hospital Huzhou University, Huzhou, China; ^4^ Department of Physical Examination Center, Zhejiang Xinda Hospital, Huzhou, China

**Keywords:** oral cancer, cause of death, survival, SEER, standardized mortality ratios (SMRs)

## Abstract

**Background:**

Nowadays, the number of oral cancer survivors is increasing, emphasizing the importance of thoroughly understanding diverse causes of death in oral cancer survivors. Our study aimed to investigate the distribution of causes of death after oral cancer diagnosis.

**Methods:**

Eligible patients were identified between 2004 and 2015 from the Surveillance, Epidemiology, and End Results (SEER) database. We calculated the number of deaths in different demographic and clinicopathological variables during each follow-up period. Standardized mortality ratios(SMRs) were generated for each cause of death after oral cancer diagnosis.

**Results:**

A total of 30538 patients diagnosed with oral cancer were included, and 17654 deaths were reported during follow-up period. 27.08% of deaths were caused by non-caner reasons. The proportion of non-cancer related deaths increased with the extension of survival time, and non-cancer death accounted for 57.93% of all deaths when followed up more than 10 years. The most common non-cancer cause of death was cardiovascular disease (SMR 4.68#; 95%CI 4.46-4.92).

**Conclusions:**

Non-cancer causes of death should not be ignored in oral cancer patients. For oral cancer survivors, multidisciplinary follow-up strategy should be recommended to achieve longer survival time.

## 1 Introduction

Oral cancer(OC) is one of the most commonly occurring malignancies in head and neck, depicted by diverse geographic distribution in its incidence and prevalence. In 2024, there will be an estimated number of 36620 newly diagnosed cases and 7930 estimated deaths in the United States(US) (1). Oral squamous cell carcinoma(OSCC) dominates all oral cancer cases. Behaviors identified as risk factors increase the likelihood of oral cancer, such as betel nut chewing, tobacco use, excessive alcohol consumption, and poor oral hygiene (2–6).

Due to early diagnosis and advances in treatment effectiveness, the number of cancer survivors is remarkably increasing. It is estimated that there are more than 300000 males survivors with a previous diagnosis of oral and pharynx cancer in US (7). Oral cancer and its treatments affect major structures involved in eating, swallowing, breathing, and communicating. Besides, non-cancer related comorbidity also complicates the survival of oral cancer patients. Therefore, understanding the real causes of death in oral cancer patients could help optimize health management and develop individual follow-up strategies.

Few studies have focused on the causes of death in oral cancer patients, especially for non-cancer related reasons. In this study, we provide a population-based analysis concentrating on each cause of death among oral cancer patients. We related different causes of death to time periods with respect to demographic and clinicopathological characteristics. Further, the risk of death from each cause in oral cancer was compared with that of the general population.

## 2 Materials and methods

### 2.1 Data source

This was a retrospective, observational cohort study. To carry out our study, we used the National Cancer Institute’s Surveillance, Epidemiology, and End Results(SEER) database. Before the use of SEER database, we registered in the SEER website with our email address, completed the application form and submitted the SEER research data use agreement form. The SEER program processed our request and we received an email with the SEER*Stat account. Then we download and installed SEER*Stat software and logged on SEER*Stat to request the data. Data was extracted from the database SEER 17 registries, Nov 2022 submission(2000-2020) for SMRs, which covers approximately 26.5% of the US population.

### 2.2 Patients

Patients diagnosed with oral cancer between 2004 and 2015 in SEER database were included. Only first primary malignancies were selected. We identified patients with a primary site in the tongue(C01.9-02.9), floor of mouth(C04.0-04.9), gum and other mouth(C03.0-03.9, C05.0-05.9, C06.0-06.9). Cases diagnosed simply through death certificates or autopsy were excluded. In addition, we also excluded patients with unknown stage information, vital status, reasons of death and follow-up time.

### 2.3 Study variables

The following demographic and clinicopathological information was gained from SEER database, including sex(male or female), age(<65 or ≥65), race(white, black or other), marital status(married, never married or other), year of diagnosis(2004-2007, 2008-2011 or 2012-2015), primary site(tongue, floor of mouth, or gum and other mouth), stage(I/II, III or IV), surgery(yes, no or unknow), radiation(yes, or no/unknow), and chemotherapy(yes, or no/unknow).

### 2.4 Outcome assessments

For included patients with oral cancer in our study, we computed the number of deaths after diagnosis with consideration to demographic and clinicopathological covariates during each follow-up period(<1 year, 1-5 years, 5-10 years and >10 years) and all follow-up time. The SEER cause of death record was categorized by the International Statistical Classification of Diseases and Related Health Problems 10th Revision(ICD-10). The SEER cause of death record guideline is shown in **Supplementary File 1**.

### 2.5 Statistical analysis

Standardized mortality ratios (SMRs) with 95% confidence intervals(CIs) were generated for each cause of death via SEER*Stat(version 8.4.3). SMRs were defined as the observed-to-expected number of deaths ratios. The “observed” represents the number of oral cancer patients who died from any defined cause in a specific period, while the “expected” represents the number of people who are expected to die from the same cause within the same timeframe in a standard population, and the standard population was adjusted based on age, sex, race and calendar year. The SMRs in our study provides the excess mortality of a specific cause after oral cancer diagnosis relatively to the general mortality in US. When observed mortality events attributed to a specific cause in oral cancer patients were significantly higher than expected mortality events for the same cause in the standard population, increased mortality risk was considered. Statistical tests were two-sided, and a *p*-value < 0.05 was recognized to be statistically significant.

## 3 Results

### 3.1 Baseline characteristics

In our study, a total of 30538 patients diagnosed with oral cancer were reviewed between 2004 and 2015 in SEER database. The number of male patients(69.85%) was nearly 2.3-fold that of the female patients(30.15%). Most oral cancer patients were aged <65 years(60.50%). The majority of the patients were white(85.74%). More than half of the included patients were married(53.11%). The proportion of patients diagnosed with stage I/II(35.27%) and stage IV(49.50%) oral cancer outnumbered, while the proportion of patients diagnosed with stage III(15.23%) oral cancer was in the minority. As to treatment regimes, surgery(58.72%) or radiotherapy(62.24%) was performed in more than half of patients, while chemotherapy(44.70%) was performed in less than half of patients.

During follow-up period, 17654(57.81%) oral cancer patients died. 5085(28.80%) deaths were occurred within 1 year of diagnosis, 8223(46.58%) deaths occurred from 1 to 5 years, 3224(18.26%) deaths occurred from 5 to 10 years, and 1122(6.36%) deaths occurred after 10 years. In addition, the proportion of cancer-related deaths in oral cancer patients showed a decreasing tendency, but the proportion of non-cancer deaths increased over time. Besides, cardiovascular disease was the most common non-cancer cause of deaths.

**Table 1** shows the baseline characteristics of oral cancer patients included in our study. **Table 2** shows observed deaths and SMRs for causes of death after diagnosis of oral cancer. **Figure 1** shows percentage of each cause of death after oral cancer diagnosis within each follow-up period.

**Table 1:** Baseline characteristics of patients with oral cancer.

Group	All Patients Diagnosed With Oral Cancer, No. (%)	Time of Death After Diagnosis,No. (%)				
		All Years	<1 year	1 to <5 years	5 to <10 years	≥10 years
All patients	30538 (100%)	17654 (100%)	5085 (100%)	8223 (100%)	3224 (100%)	1122 (100%)
Sex						
Male	21330 (69.85%)	12256 (69.42%)	3366 (66.19%)	5853 (71.18%)	2279 (70.69%)	758 (67.56%)
Female	9208 (30.15%)	5398 (30.58%)	1719 (33.81%)	2370 (28.82%)	945 (29.31%)	364 (32.44%)
Age, years						
<65	18474 (60.50%)	9026 (51.13%)	2361 (46.43%)	4461 (54.25%)	1574 (48.82%)	630 (56.15%)
≥65	12064 (39.50%)	8628 (48.87%)	2724 (53.57%)	3762 (45.75%)	1650 (51.18%)	492 (43.85%)
Race						
White	26183 (85.74%)	14897 (84.38%)	4106 (80.75%)	6929 (84.26%)	2863 (88.80%)	999 (89.04%)
Black	2347 (7.69%)	1749 (9.91%)	667 (13.12%)	800 (9.73%)	209 (6.48%)	73 (6.51%)
Other	2008 (6.58%)	1008 (5.71%)	312 (6.14%)	494 (6.01%)	152 (4.71%)	50 (4.46%)
Marital status						
Married	16220 (53.11%)	8153 (46.18%)	1939 (38.13%)	3901 (47.44%)	1677 (52.02%)	636 (56.68%)
Never married	5526 (18.10%)	3529 (19.99%)	1167 (22.95%)	1650 (20.07%)	533 (16.53%)	179 (15.95%)
Other	8792 (28.79%)	5972 (33.83%)	1979 (38.92%)	2672 (32.49%)	1014 (31.45%)	307 (27.36%)
Year of diagnosis						
2004-2007	8575 (28.08%)	6091 (34.50%)	1578 (31.03%)	2482 (30.18%)	1196 (37.10%)	835 (74.42%)
2008-2011	10167 (33.29%)	6024 (34.12%)	1618 (31.82%)	2776 (33.76%)	1343 (41.66%)	287 (25.58%)
2012-2015	11796 (38.63%)	5539 (31.38%)	1889 (37.15%)	2965 (36.06%)	685 (21.25%)	0 (0.00%)
Primary site						
Tongue	20847 (68.27%)	10858 (61.50%)	3017 (59.33%)	5118 (62.24%)	1982 (61.48%)	741 (66.04%)
Floor of mouth	3226 (10.56%)	2302 (13.04%)	640 (12.59%)	1077 (13.10%)	448 (13.90%)	137 (12.21%)
Gum and other mouth	6465 (21.17%)	4494 (25.46%)	1428 (28.08%)	2028 (24.66%)	794 (24.63%)	244 (21.75%)
Stage						
I/II	10771 (35.27%)	5255 (29.77%)	691 (13.59%)	2564 (31.18%)	1483 (46.00%)	517 (46.08%)
III	4652 (15.23%)	2743 (15.54%)	747 (14.69%)	1335 (16.23%)	483 (14.98%)	178 (15.86%)
IV	15115 (49.50%)	9656 (54.70%)	3647 (71.72%)	4324 (52.58%)	1258 (39.02%)	427 (38.06%)
Surgery						
Yes	17933 (58.72%)	9681 (54.84%)	2007 (39.47%)	4858 (59.08%)	2101 (65.17%)	715 (63.73%)
No	12457 (40.79%)	7877 (44.62%)	3043 (59.84%)	3321 (40.39%)	1113 (34.52%)	400 (35.65%)
Unknow	148 (0.48%)	96 (0.54%)	35 (0.69%)	44 (0.54%)	10 (0.31%)	7 (0.62%)
Radiation						
Yes	19006 (62.24%)	11281 (63.90%)	3019 (59.37%)	5672 (68.98%)	1925 (59.71%)	665 (59.27%)
No/Unknow	11532 (37.76%)	6373 (36.10%)	2066 (40.63%)	2551 (31.02%)	1299 (40.29%)	457 (40.73%)
Chemotherapy						
Yes	13651 (44.70%)	7932 (44.93%)	2216 (43.58%)	3968 (48.25%)	1292 (40.07%)	456 (40.64%)
No/unknow	16887 (55.30%)	9722 (55.07%)	2869 (56.42%)	4255 (51.75%)	1932 (59.93%)	666 (59.36%)

**Table 2:** Observed deaths and SMRs for causes of death after diagnosis of oral cancer.

	Timing of Death After Diagnosis									
	All years		<1 year		1 to <5 years		5 to <10 years		≥10 years	
	No. (%)	SMR (95% CI)	No. (%)	SMR (95% CI)	No. (%)	SMR (95% CI)	No. (%)	SMR (95% CI)	No. (%)	SMR (95% CI)
Cause of Death	17654 (100%)	4.36# (4.29-4.42)	5085 (100%)	10.79# (10.50-11.09)	8223 (100%)	4.85# (4.74-4.95)	3224 (100%)	2.35# (2.27-2.43)	1122 (100%)	2.19# (2.07-2.32)
All Malignant Cancers	12796 (72.48%)	13.18# (12.95-13.41)	4220 (82.99%)	35.68# (34.61-36.77)	6422 (78.10%)	15.28# (14.90-15.65)	1687 (52.33%)	5.27# (5.02-5.53)	467 (41.62%)	4.15# (3.79-4.55)
Oral Cavity and Pharynx	7102 (40.23%)	399.97# (390.73-409.39)	2706 (53.22%)	1324.37# (1274.93-1375.22)	3533 (42.96%)	465.70# (450.47-481.31)	684 (21.22%)	114.31# (105.90-123.21)	179 (15.95%)	83.54# (71.75-96.71)
Other Oral Cavity and Pharynx	552 (3.13%)	181.19# (166.39-196.95)	190 (3.74%)	477.98# (412.43-550.99)	267 (3.25%)	195.30# (172.58-220.19)	74 (2.30%)	76.40# (59.99-95.92)	21 (1.87%)	67.00# (41.48-102.42)
Digestive System	521 (2.95%)	1.99# (1.82-2.17)	68 (1.34%)	2.19# (1.70-2.78)	243 (2.96%)	2.16# (1.90-2.45)	149 (4.62%)	1.71# (1.45-2.01)	61 (5.44%)	1.97# (1.51-2.53)
Respiratory System	1199 (6.79%)	4.33# (4.09-4.58)	195 (3.83%)	5.45# (4.72-6.28)	593 (7.21%)	4.82# (4.44-5.22)	325 (10.08%)	3.67# (3.28-4.09)	86 (7.66%)	2.91# (2.33-3.60)
Bones and Joints	175 (0.99%)	93.52# (80.18-108.45)	90 (1.77%)	440.42# (354.15-541.36)	65 (0.79%)	83.90# (64.75-106.94)	15 (0.47%)	23.22# (13.00-38.31)	5 (0.45%)	20.29# (6.59-47.36)
Soft Tissue including Heart	18 (0.10%)	2.80# (1.66-4.43)	4 (0.08%)	5.32# (1.45-13.63)	5 (0.06%)	1.81 (0.59-4.23)	4 (0.12%)	1.86 (0.51-4.77)	5 (0.45%)	6.58# (2.14-15.36)
Skin excluding Basal and Squamous	517 (2.93%)	22.76# (20.84-24.81)	165 (3.24%)	61.23# (52.25-71.32)	286 (3.48%)	29.05# (25.78-32.62)	52 (1.61%)	6.89# (5.15-9.04)	14 (1.25%)	5.32# (2.91-8.93)
Breast/Genital/Urinary/Endocrine System	156 (0.88%)	4.85# (4.12-5.68)	13 (0.26%)	3.20# (1.70-5.46)	62 (0.75%)	3.61# (2.77-4.63)	63 (1.95%)	6.89# (5.30-8.82)	18 (1.60%)	10.17# (6.02-16.07)
Eye and Orbit/Brain and Other Nervous System	31 (0.18%)	9.77# (6.64-13.86)	6 (0.12%)	11.86# (4.35-25.80)	13 (0.16%)	6.80# (3.62-11.62)	11 (0.34%)	16.22# (8.10-29.03)	1 (0.09%)	12.89 (0.33-71.84)
lymph/Blood	110 (0.62%)	4.95# (4.07-5.96)	10 (0.20%)	3.95# (1.89-7.26)	44 (0.54%)	4.52# (3.29-6.07)	38 (1.18%)	4.66# (3.30-6.39)	18 (1.60%)	9.88# (5.86-15.61)
Miscellaneous Malignant Cancer	2967 (16.81%)	40.01# (38.58-41.48)	963 (18.94%)	107.60# (100.91-114.62)	1578 (19.19%)	49.53# (47.12-52.04)	346 (10.73%)	14.09# (12.64-15.65)	80 (7.13%)	9.10# (7.22-11.33)
In situ, benign or unknown behavior neoplasm	78 (0.44%)	3.08# (2.44-3.85)	23 (0.45%)	7.83# (4.97-11.75)	32 (0.39%)	3.00# (2.05-4.24)	18 (0.56%)	2.10# (1.25-3.32)	5 (0.45%)	1.60 (0.52-3.73)
Noncancer	4780 (27.08%)	4.93# (4.79-5.07)	842 (16.56%)	6.92# (6.46-7.41)	1769 (21.51%)	3.84# (3.66-4.02)	1519 (47.12%)	4.92# (4.68-5.18)	650 (57.93%)	8.26# (7.64-8.92)
Infections	269 (1.52%)	9.56# (8.45-10.78)	83 (1.63%)	18.89# (15.05-23.42)	98 (1.19%)	7.03# (5.71-8.57)	71 (2.20%)	8.61# (6.72-10.86)	17 (1.52%)	10.91# (6.35-17.46)
Diabetes Mellitus	123 (0.70%)	0.97 (0.81-1.16)	27 (0.53%)	1.84# (1.21-2.67)	47 (0.57%)	0.89 (0.65-1.18)	35 (1.09%)	0.81 (0.57-1.13)	14 (1.25%)	0.87 (0.48-1.46)
Alzheimers (ICD-9 and 10 only)	137 (0.78%)	0.95 (0.80-1.12)	7 (0.14%)	0.48# (0.19-0.99)	44 (0.54%)	0.78 (0.57-1.05)	56 (1.74%)	1.08 (0.82-1.40)	30 (2.67%)	1.41 (0.95-2.01)
Cardiovascular Disease	1581 (8.96%)	4.68# (4.46-4.92)	303 (5.96%)	6.65# (5.92-7.44)	605 (7.36%)	3.68# (3.39-3.99)	474 (14.70%)	4.59# (4.18-5.02)	199 (17.74%)	8.17# (7.08-9.39)
Cerebrovascular Diseases	325 (1.84%)	1.56# (1.40-1.74)	45 (0.88%)	1.82# (1.33-2.43)	109 (1.33%)	1.26# (1.03-1.52)	117 (3.63%)	1.67# (1.38-2.00)	54 (4.81%)	2.04# (1.53-2.66)
Pneumonia and Influenza	196 (1.11%)	2.35# (2.03-2.71)	34 (0.67%)	3.31# (2.29-4.62)	72 (0.88%)	2.03# (1.59-2.56)	61 (1.89%)	2.20# (1.68-2.83)	29 (2.58%)	2.94# (1.97-4.22)
Chronic Obstructive Pulmonary Disease and Allied Cond	446 (2.53%)	1.79# (1.63-1.97)	60 (1.18%)	2.10# (1.60-2.70)	153 (1.86%)	1.45 (1.23-1.70)	155 (4.81%)	1.83# (1.56-2.15)	78 (6.95%)	2.54# (2.01-3.17)
Stomach and Duodenal Ulcers	16 (0.09%)	3.13# (1.79-5.08)	6 (0.12%)	9.76# (3.58-21.24)	5 (0.06%)	2.32 (0.75-5.42)	4 (0.12%)	2.34 (0.64-5.99)	1 (0.09%)	1.58 (0.04-8.79)
Chronic Liver Disease and Cirrhosis	134 (0.76%)	2.31# (1.94-2.74)	19 (0.37%)	2.79# (1.68-4.35)	73 (0.89%)	2.89# (2.26-3.63)	34 (1.05%)	1.76# (1.22-2.47)	8 (0.71%)	1.21 (0.52-2.39)
Nephritis/Nephrotic Syndrome/Nephrosis	79 (0.45%)	1.05 (0.83-1.31)	11 (0.22%)	1.23 (0.61-2.20)	27 (0.33%)	0.85 (0.56-1.23)	30 (0.93%)	1.19 (0.80-1.70)	11 (0.98%)	1.19 (0.59-2.13)
Pregnancy/Childbirth/Puerperium	1 (0.01%)	15.90 (0.40-88.57)	0 (0)	0.00 (0.00-362.41)	1 (0.01%)	31.49 (0.80-175.42)	0 (0)	0.00 (0.00-208.41)	0 (0)	0.00 (0.00-1129.51)
Congenital Anomalies	10 (0.06%)	2.36# (1.13-4.34)	1 (0.02%)	1.92 (0.05-10.70)	3 (0.04%)	1.61 (0.33-4.69)	3 (0.09%)	2.17 (0.45-6.33)	3 (0.27%)	6.43# (1.33-18.79)
Symptoms,signs and ill-defined conditions	91 (0.52%)	2.15# (1.73-2.64)	20 (0.39%)	3.75# (2.29-5.79)	40 (0.49%)	2.16# (1.54-2.94)	22 (0.68%)	1.59 (1.00-2.41)	9 (0.80%)	1.95 (0.89-3.70)
Accidents and Adverse Effects	255 (1.44%)	1.83# (1.61-2.07)	34 (0.67%)	2.12# (1.47-2.96)	112 (1.36%)	1.89# (1.56-2.27)	77 (2.39%)	1.64# (1.29-2.05)	32 (2.85%)	1.88# (1.29-2.66)
Suicide and Self-Inflicted Injury	118 (0.67%)	2.94# (2.43-3.52)	32 (0.63%)	6.40# (4.37-9.03)	44 (0.54%)	2.43# (1.77-3.26)	37 (1.15%)	2.88# (2.02-3.96)	5 (0.45%)	1.19 (0.39-2.77)
Homicide and Legal Intervention	7 (0.04%)	1.20 (0.48-2.47)	0 (0)	0.00 (0.00-4.32)	6 (0.07%)	2.21 (0.81-4.81)	1 (0.03%)	0.58 (0.01-3.21)	0 (0)	0.00 (0.00-7.07)
Other Cause of Death	992 (5.62%)	1.46# (1.37-1.55)	160 (3.15%)	2.34# (1.99-2.73)	330 (4.01%)	1.26# (1.13-1.40)	342 (10.61%)	1.39# (1.24-1.54)	160 (14.26%)	1.55# (1.32-1.81)

CI, confidence interval; SMR, standardized mortality ratio.

#*p*<0.05.

**Figure 1:**
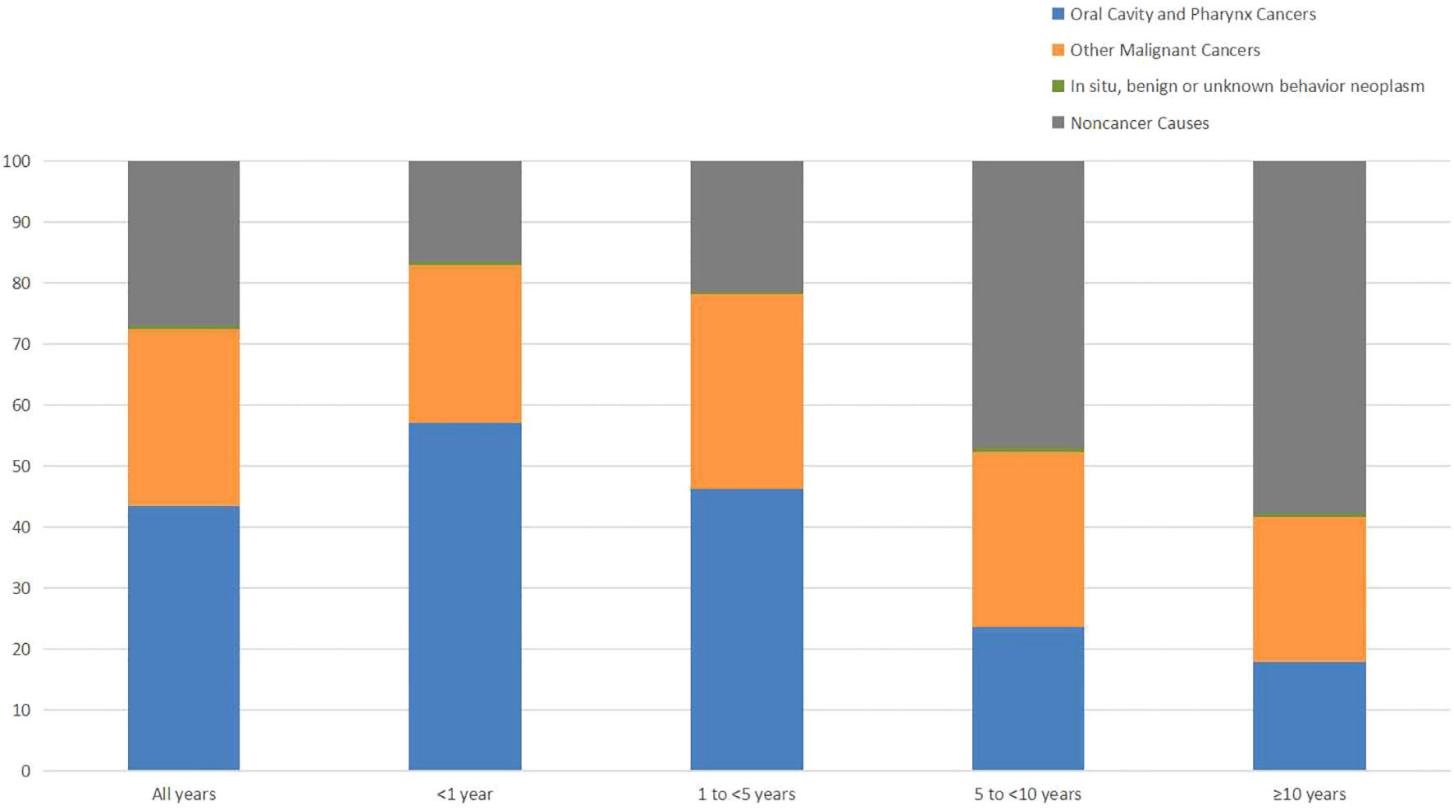
Percentage of causes of death after oral cancer diagnosis within each follow-up period.

### 3.2 Cause of death within 1 year following OC diagnosis

Within the first year following oral cancer diagnosis, 5085 patients died. The majority of deaths were caused by cancer-related reasons(82.99%), while non-cancer reasons were only a small fraction(16.56%). Oral cavity and pharynx cancers(56.95%) were the most common causes of cancer-related deaths, followed by miscellaneous malignant cancer(18.94%). Cardiovascular disease(5.96%) was the leading non-cancer related cause of deaths. Patients with oral cancer had a higher risk dying from infections than the general population(SMR 18.89#; 95%CI 15.05-23.42). Additionly, the risk of dying from digestive ulcers(SMR 9.76#; 95%CI 3.58-21.24) and cardiovascular disease(SMR 6.65#; 95%CI 5.92-7.44) also increased.

### 3.3 Cause of death within 1-5 years following OC diagnosis

A total of 8223 patients died within 1-5 years following oral cancer diagnosis, 6422(78.10%) patients died of cancer causes, and 1769(21.51%) patients died of non-cancer causes. Oral cavity and pharynx cancers(46.21%) remained to be the most frequent cancer-related cause of deaths, while miscellaneous malignant cancer(19.19%) dominated other caner-related cause of death. Besides, cardiovascular disease(7.36%) continued to be the most common non-cancer related cause of deaths. The risk of dying from cardiovascular disease(SMR 3.68#; 95%CI 3.39-3.99) was highest in non-cancer causes.

### 3.4 Cause of death within 5-10 years following OC diagnosis

A total of 3224 patients died within 5-10 years following oral cancer diagnosis, 1687(52.33%) patients died of cancer causes, and 1519(47.12%) patients died of non-cancer causes. Apart from oral cavity and pharynx cancers(23.51%), miscellaneous malignant cancer(10.73%) and respiratory system cancer(10.08%) were the commonest cancer-related causes. For non-cancer causes, leading causes were cardiovascular disease(14.70%), other cause of death(10.61%), and chronic obstructive pulmonary disease(4.81%). The risk of oral cancer patients dying from infections(SMR 8.61#; 95%CI 6.72-10.86) and cardiovascular disease(SMR 4.59#; 95%CI 4.18-5.02) increased than the general population.

### 3.5 Cause of death after 10 years following OC diagnosis

After 10 years following oral cancer diagnosis, a total of 1122 patients died. The percentage of patients died of cancer-related causes(41.62%) decreased, but the number of patients died of non-cancer causes(57.93%) increased. Oral cavity and pharynx cancers(17.83%) still dominated cancer causes, followed by respiratory system cancers(7.66%) and miscellaneous malignant cancer(7.13%). In addition, cardiovascular disease(17.74%) continued to be the most common cause of non-cancer related deaths. The risks of oral cancer patients dying from infections(SMR 10.91#; 95%CI 6.35-17.46), cardiovascular disease(SMR 8.17#; 95%CI 7.08-9.39), and congenital anomalies(SMR 6.43#; 95%CI 1.33-18.79) were 6 times higher than what expected in the general population.

### 3.6 Subgroup analysis

Male and female oral cancer patients had a similar risk of all causes of death and non-cancer related causes of death (**Supplementary Tables S1**, **S2**). Oral cancer patients aged < 65 years old had a higher risk of cancer-related and non-cancer related death than that of patients aged≥ 65 years old (**Supplementary Tables S3**, **S4**). Patients of black had greater risk of death than patients of white or other races, regardless of caner or non-cancer causes (**Supplementary Tables S5–S7**). Patients never married had a higher risk of death than patients married or in other marital status, no matter it was a cancer or non-cancer factor (**Supplementary Tables S8–S10**). Patients diagnosed recently showed an increase in non-cancer related causes of death (**Supplementary Tables S11–S13**). Patients with oral cancer occurred in floor of mouth had a higher risk of death from cancer or non-cancer causes (**Supplementary Tables S14–S16**). Patients diagnosed with stage IV oral cancer had a greater risk of death for both cancer and non-cancer causes than patients diagnosed with I-III oral cancer (**Supplementary Tables S17–S19**). Patients underwent surgery had a lower risk of cancer and non-cancer causes of death (**Supplementary Tables S20–S22**). Risk of non-cancer related causes of death was higher in patients who received radiotherapy or chemotherapy (**Supplementary Tables S23–S26**).

## 4 Discussion

With improvements in early detection, anti-tumor treatments and supportive care, the number of oral cancer survivors is increasing. Understanding the real causes of death is vital for oral cancer patients to optimize follow-up strategies. In our study, we analyzed causes of death of 30538 patients diagnosed with oral cancer from 2004 to 2015 in SEER database. A total of 17654 patients died during follow-up period, and the mortality rate reached 57.81%. More than half of the deaths were causes by other types of cancer and non-cancer causes. Cancer-related causes were responsible for the majority of deaths in the initial phase after diagnosis. But with the extension of survival time, the percentage of non-cancer related deaths gradually increased.

Second primary cancer is a leading long-term cause of death for oral cancer survivors. Apart from oral and pharynx cancer, we observed 5145 deaths due to other malignant cancer, of which miscellaneous malignant cancer and respiratory system cancer were the most frequent. A previous study confirmed that patients with oral squamous cell cancer had an 85% excess risk of growing second primary cancer compared with the incidence rate in Finnish population (8). And respiratory organs, intrathoracic organs and digestive organs were the commonest site for second primary cancer (8). Another study indicated that oral cancer patients had a substantially higher risk for second primary hypopharyngeal and esophageal cancer and had poor prognosis (9). Besides, a population-based, retrospective study found a noteworthy increased rate of second primary cancer after treatment of oral squamous cell cancer, especially in head and neck region and in lungs (10). Several factors may influence the development of second primary cancer, such as gene mutations, environmental exposures, and living habits (11–14). Anti-tumor treatments also influence the development of second primary cancer, such as radiation exposure (15).

The most common non-cancer cause of death was cardiovascular disease. Cancers have been found to be strongly associated with cardiovascular disease (16, 17). A representative cohort study in Taiwan discovered that cancer patients had a significantly greater risk of fatal or non-fatal cardiovascular disease (18). Cardio-oncology was proposed to focus on diagnosis, treatment and prevention of cardiovascular consequences of cancer and its treatment. Betel quid chewing is one of the major factors of developing oral cancer. Previous studies concluded that betel quid chewers would experience much more incidence of cardiovascular disease (19–21). Besides, tobacco was recognized to be the risk factor for oral cancer and cardiovascular disease.

The risk of death from infectious diseases in oral cancer patients was significantly increased. Infection, a preventable cause, was thought to be related to the development of oral cancer (22–24). Among oral cancer survivors, as a consequence of disease and its treatments, infectious conditions of any nature, viral, bacterial, and fungal were frequent (25). Mouth infections can affect the prognosis of patients with oral cancer. Fungal were the most common opportunistic infections. Neutropenia caused by cancer and its treatments would promote the invasion of Candida species and lead to clinical debilitating infections (26). Besides, mucosal damage resulting from oral cancer increased the risk of infectious complications. Moreover, aspiration pneumonia and surgical site infection remained to be a critical problems for surgery-induced complications (27).

In addition, the risk of dying from stomach and duodenal ulcers in oral cancer patients was higher than the general population, especially in the first year of diagnosis. Radiotherapy is widely used for head and neck cancer patients. In 9388 head and neck cancer patients treated with radiotherapy, the percentage of comorbidity about peptic ulcer diseases was 6.8% (28). Besides, in 232 oral cancer patients post major surgical intervention with or without adjuvant therapy, peptic ulcer(8.6%) was the most common comorbidity (29).

Surgery is still the primary therapeutic option for oral cancer. The goal of surgery is complete resection of tumor with an adequate margin. Margin status diagnosis, significantly influences the occurrence of tumor and quality of life, should be accessed cautiously (30, 31). The surgical approach in oral cancer has evolved over the years. A retrospective study compared patients treated by invasive surgery with those treated by less invasive approach and concluded that the invasive approach does not correlate to better survival or locoregional control (32).

With the progress of radiation techniques, modern radiotherapy aims to improve therapeutic efficacy and side effects. Radiotherapy is recommended for parts of postoperative oral cancer patients or patients who are not suitable for surgery. Intensity-modulated radiotherapy(IMRT) or volumetric intensity-modulated arc therapy(VMAT), administering high doses of radiation to a small area of oral cavity of many critical structures, was reported to reduce radiation-related toxicity, such as xerostomia, dysphagia, severe mucositis (33–35). Besides, in head and neck cancers, IMRT and VMAT reduce the possibility of second primary cancer within the in-filed volume compared to conventional radiotherapy (35). Besides, the risk of radiation-induced carcinogenesis decreases for VMAT, as a result of the reduction of monitor unit and the whole-body integral dose (36).

Chemotherapy can be used as postoperative adjuvant therapy, primary treatment when surgery is not possible, concurrent chemoradiotherapy, or palliative therapy. Adverse effects vary depending on chemotherapeutic agents. Agents commonly used in oral cancer include cisplatin, 5-fluorouracil, docetaxel, paclitaxel and gemcitabine. Renal toxicity is a well-known adverse effect of cisplatin, and adequate hydration is required during cisplatin injection. Cardiotoxicity is a severe adverse effect of 5-fluorouracil following cancer treatment (37). Cetuximab, a monoclonal antibody against epidermal growth factor receptor(EGFR), has an established role in oral cancer. But cetuximab can cause severe skin, mucosal, gastrointestinal and kidney toxicity. New inhibitors like nimotuzumab, have been developed with higher EGFR affinity and fewer side effects (38).

However, our study has several limitations that should be addressed. First, our study was retrospective, and bias was inevitable, particularly in the selection of patients and the accuracy of recorded data. Second, the prevalence of oral cancer is depicted by diverse geographic disparity, with common occurrence in South Asia countries. But the majority participants in our study were white. Also, the study may not capture the full spectrum of oral cancer patients because of our exclusion criteria. Whether our finding can be applied to all oral cancer patients need further investigation. Furthermore, the administrative data in SEER database may not include detailed clinical information, such as comorbid states, treatment strategies and complications, which could be relevant to the outcomes in our study.

## 5 Conclusions

In conclusion, with the extension of survival, the number of oral cancer patients dying from non-cancer related causes increased. Cardiovascular disease was the most common non-cancer cause of death in oral cancer patients. Besides, the risk of dying from infections, cardiovascular disease or peptic ulcers was higher in oral cancer patients. Therefore, being aware of non-cancer risk factors and optimizing follow-up strategies are crucial to achieve longer survival time and better quality of life for oral cancer patients.

## 6 Future directions

For oral cancer patients, our study highlighted that attention should be paid not only to anti-tumor therapy but also to the occurrence of other risks. As the survival time prolonged, the problem of second primary cancer and non-cancer related deaths emerged. Future studies are encouraged to explore second primary cancer of oral cancer survivors, particularly focusing on the prevention of second primary cancer. In addition, newly developing subjects, such as cardio-oncology, are required to research, treat and prevent non-cancer diseases for oral cancer patients. Furthermore, oral cancer patients in our study were diagnosed between 2004 and 2015 in SEER database. We need further research about causes of death for oral cancer patients diagnosed recently, due to the rapidly improved treatments.

## Data Availability

The datasets presented in this study can be found in online repositories. The names of the repository/repositories and accession number(s) can be found below: https://seer.cancer.gov/seerstat/software.
