# Supplementary material for: Causes of death after oral cancer diagnosis: a population based study
Source: Front Oncol. 2024 Nov 12;14:1481688. doi: 10.3389/fonc.2024.1481688 (PMC11588711; doi:10.3389/fonc.2024.1481688)
Supplement: Supplementary file 1 [file DataSheet1.zip › Supplementary files/Supplementary file 2.pdf]

伦理证明

项目类型	论文
项目名称	Causes of Death after Oral Cancer Diagnosis: A Population Based Study
负责人	蒋震宇

伦理委员会审阅意见：

该论文经审阅未发现对病人的权益产生不利的影响，未泄露病人的隐私和身份信息。

湖州市第一人民医院

医学科研与临床试验伦理委员会

2024年10月7日
